# Calcium and Spike Timing-Dependent Plasticity

**DOI:** 10.3389/fncel.2021.727336

**Published:** 2021-09-20

**Authors:** Yanis Inglebert, Dominique Debanne

**Affiliations:** ^1^UNIS, UMR1072, INSERM, Aix-Marseille University, Marseille, France; ^2^Department of Pharmacology and Therapeutics and Cell Information Systems, McGill University, Montreal, QC, Canada

**Keywords:** synaptic plasticity, synapse, STDP, hippocampus, learning, memory, calcium

## Abstract

Since its discovery, spike timing-dependent synaptic plasticity (STDP) has been thought to be a primary mechanism underlying the brain’s ability to learn and to form new memories. However, despite the enormous interest in both the experimental and theoretical neuroscience communities in activity-dependent plasticity, it is still unclear whether plasticity rules inferred from *in vitro* experiments apply to *in vivo* conditions. Among the multiple reasons why plasticity rules *in vivo* might differ significantly from *in vitro* studies is that extracellular calcium concentration use in most studies is higher than concentrations estimated *in vivo*. STDP, like many forms of long-term synaptic plasticity, strongly depends on intracellular calcium influx for its induction. Here, we discuss the importance of considering physiological levels of extracellular calcium concentration to study functional plasticity.

## Introduction

Spike timing-dependent plasticity (STDP) is a form of long-term synaptic modification thought to constitute a mechanism underlying formation of new memories. The polarity of synaptic modifications is controlled by the relative timing between pre- and post-synaptic action potentials (APs; Dan and Poo, 2004; Feldman, 2012). Following the Konorski-Hebb principle (Konorski, 1948; Hebb, 1949), timing-dependent long-term synaptic potentiation (t-LTP) in hippocampal and neocortical pyramidal neurons, results from the temporal conjunction of synaptic activity followed by one or more backpropagating APs in the post-synaptic cell (Gustafsson and Wigström, 1986; Markram et al., 1997; Bi and Poo, 1998; Debanne et al., 1998; Feldman, 2000). In contrast, following Stent principle (Stent, 1973), timing-dependent synaptic depression (t-LTD) is induced when synaptic activity is repeatedly preceded by one of more backpropagating action potentials (Debanne et al., 1994, 1996a, 1998; Markram et al., 1997; Bi and Poo, 1998; Feldman, 2000). It is important to note that t-LTP and t-LTD have been reported in early studies when two synaptic inputs, namely a weak input producing a subthreshold response and a strong input producing an action potential were paired with positive or negative delays (Baranyi and Fehér, 1981; Levy and Steward, 1983; Stanton and Sejnowski, 1989).

## STDP and Calcium

At excitatory synapses, the amplitude of post-synaptic calcium influx determines the orientation of plasticity towards synaptic potentiation or depression (Artola et al., 1990). The better demonstration for that is provided by the fact that buffering post-synaptic calcium with BAPTA prevents the induction of both LTP and LTD (Debanne et al., 1994; Nevian and Sakmann, 2006). In a similar way, uncaging of calcium in CA1 pyramidal cells selectively induces LTP or LTD depending on the magnitude of calcium influx (Yang et al., 1999). In Hebbian STDP, the correlation between an EPSP and the backpropagated action potential (bAP) corresponding to a pre-before-post pairing that leads to t-LTP, induces large calcium entry (Koester and Sakmann, 1998). In contrast, post-before-pre pairing that induces t-LTD, comparatively produces a weaker calcium entry (Koester and Sakmann, 1998). Induction of t-LTP involves several mechanisms: (1) removal of the magnesium block from the NMDA receptor (Kampa et al., 2004); (2) inactivation of A-type current and activation of sodium channels to improve signal propagation (Hoffman et al., 1997; Stuart and Häusser, 2001); and (3) AMPA receptor depolarization to boost NMDA receptor calcium signal (Fuenzalida et al., 2010; Holbro et al., 2010). However, the NMDA receptor is the major player as its blockade (by perfusion of MK801 in the post-synaptic neuron for example) prevents LTP induction. Expression of t-LTP requires kinases activation such as CaMKII to phosphorylate AMPA and NMDA receptors thereby increasing their conductance (Otmakhova et al., 2002; Lisman et al., 2012) as well as the insertion of new AMPA receptors (Malinow and Malenka, 2002). In t-LTD, low calcium entry leads to inactivation of NMDA receptors by activation of phosphatases (Rosenmund et al., 1995). These two forms of plasticity which are dependent on post-synaptic NMDA receptors are found in the hippocampus at CA3-CA1 synapses of rodents (Nishiyama et al., 2000; Andrade-Talavera et al., 2016) or in the layer II/III of the cortex of rodents (Froemke et al., 2006). Some forms of LTD are also dependent on the presynaptic NMDA receptor. In the cortex or hippocampus, perfusion of MK801 in the pre-synaptic neurons prevents LTD but not LTP (Sjöström et al., 2003; Rodríguez-Moreno and Paulsen, 2008; Banerjee et al., 2009; Andrade-Talavera et al., 2016). Other forms of NMDA receptor-independent LTD expressed at hippocampal CA3-CA1 and cortical L4-L2/3 synapses, requires metabotropic glutamate receptors (mGluRs), voltage-dependent calcium channels, cannabinoid receptors and astrocytic signaling (Normann et al., 2000; Bender, 2006). In these forms of LTD, production of a retrograde messenger, the endocannabinoids (eCBs), will decrease the probability of pre-synaptic release (Bender, 2006; Chevaleyre et al., 2006). However, this simplistic view is now challenged by several studies that do not necessarily observe a correlation between calcium entry and plasticity. In layer II/III of the cortex, the same rise in post-synaptic calcium can lead to LTP or LTD (Nevian and Sakmann, 2006) and a broadening of the action potential that induce a larger calcium influx may surprisingly facilitate LTD (Zhou et al., 2005).

## NMDA Spikes

STDP relies heavily on the bAP which cannot propagate too deeply into the dendritic tree (Spruston, 2008). As a result, distal synapses require a local source of depolarization for t-LTP. Thus, dendritic NMDA receptors are an important source of calcium (Schiller and Schiller, 2001). Several studies have now shown that they are necessary for the induction of t-LTP. In the hippocampus, at mossy fibers-CA3 synapses, t-LTP can only be induced when NMDA spikes are triggered (Brandalise et al., 2016). In the cortex, at layer II/III-V distal synapses, dendritic depolarization can switch plasticity between LTD and LTP (Sjöström and Häusser, 2006). In addition, distal synapses can be cooperative. In CA1 pyramidal cells, synaptic cooperativity is observed at distal but not proximal dendritic locations following repetitive subthreshold activation of small spine clusters (Weber et al., 2016). Recently, in layer 5 pyramidal neurons, it was shown that synaptic cooperativity disrupts t-LTD and extends the temporal window for the induction of t-LTP (Tazerart et al., 2020).

## Mathematical Models of STDP Based on Calcium

Synaptic plasticity models have been first theorized by John Lisman in 1989. According to this pioneering work, high post-synaptic calcium influx represents a condition favorable to LTP induction because protein kinases are preferentially activated whereas low to moderate calcium influx induces LTD because protein phosphatases are selectively activated. Most recent mathematical models of STDP incorporate the biochemical pathways described above to link post-synaptic calcium and plasticity (Karmarkar and Buonomano, 2002; Shouval et al., 2002; Graupner and Brunel, 2010). One of the first models to investigate the role of extracellular calcium in STDP was the one developed by Graupner and Brunel (2012). By variation of the extracellular calcium, and therefore postsynaptic calcium entry, they showed that a multitude of STDP curves could be obtained in response to a simple pre-post (1:1) or post-pre protocol (1:1; Figure 1). In the most extreme cases, only LTD or LTP could be observed respectively for a very weak or very strong calcium influx. For intermediate influx, the classical curve was found with a window of t-LTD (Δt ≤ 0 ms), a window of t-LTP (Δt ≥ 0 ms) and sometimes a second window of depression for longer delays (Δt = 20–30 ms). The concentration of extracellular calcium is consequently important for the orientation of plasticity but also for its maintenance. Time scales of memory maintenance can be extended by lowering extracellular calcium concentration to *in vivo* levels (Higgins et al., 2014). Spontaneous synaptic activity is known to erase memory induced by STDP in the tadpole visual system (Zhou et al., 2003). Similar behavior has been reproduced *in silico* with a calcium-based model (Higgins et al., 2014). In the presence of background synaptic noise, synaptic changes induced by STDP disappear in a few minutes with a high concentration of calcium, whereas they last 1 hour with a physiological concentration.

**Figure 1:**
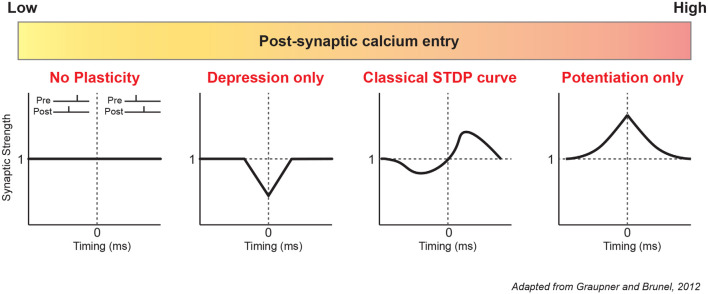
Diversity of modeled STDP curves based on calcium. According to the modeling of Graupner and Brunel (2012), post-synaptic calcium is a major determinant of plasticity. From left to right: low calcium entry causes no plasticity or only depression (*t-LTD*). Intermediate calcium entry produces the classic STDP curve (*Hebbian STDP*), with a depression window (*t-LTD)* for negative delays and a potentiation window (*t-LTP*) for positive delays. High calcium entry results in only potentiation for all delays. Note that these results were obtained with a simple 1:1 protocol (1 presynaptic, 1 postsynaptic stimulation) with a low pairing frequency of 1 Hz and 60 repetitions. STDP, Spike Timing-Dependent Plasticity.

## Role of External Calcium Level

**Figure 2:**
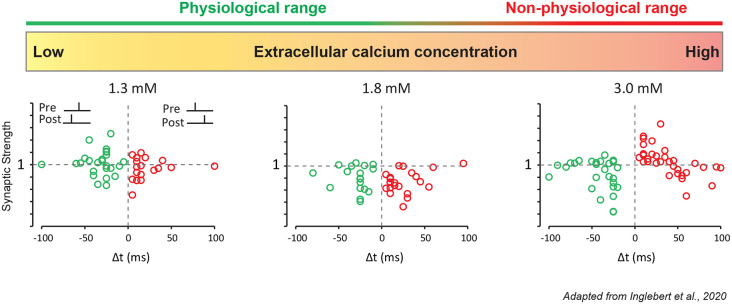
STDP under various external calcium concentrations. CA1 pyramidal neurons were recorded in a whole-cell configuration. Pre-synaptic stimulation was evoked by a stimulation electrode placed in the Schaffer collaterals. These results were obtained with a simple 1:1 protocol (1 presynaptic, 1 postsynaptic stimulation) with a low pairing frequency of 0.3 Hz and 100 repetitions for t-LTP, and 150 repetitions for t-LTD. In 1.3 mM extracellular calcium, no plasticity is induced. In 1.8 mM extracellular calcium, pre-post and post-pre protocols leads to t-LTD. In 3 mM extracellular calcium, pre-post protocol leads to t-LTP and post-pre protocol leads to t-LTD.

Most, if not all, *in vitro* studies of STDP have used non-physiological extracellular calcium concentrations ([Ca^2+^]_e_), commonly between 2 and 3 mM (Table 1). But physiological [Ca^2+^]_e_ is about 2 to 3 times lower, i.e., ranging from 1.3–1.8 mM in young rodent (Jones and Keep, 1988; Silver and Erecińska, 1990; Ding et al., 2016). In fact, past studies already showed discrepancies regarding STDP induced *in vitro*. The same protocol in the hippocampus could lead to a radically different STDP curve (Wittenberg and Wang, 2006; Campanac and Debanne, 2008) and the only obvious difference was the concentration of extracellular calcium used (2 vs. 3 mM). Recently, it has been confirmed experimentally that the overall calcium concentration has a significant effect on plasticity (Inglebert et al., 2020). At CA3-CA1 synapses, while the Ca^2+/^Mg^2+^ ratio is kept unchanged, Hebbian STDP is found for [Ca^2+^]_e_ = 3 mM while no plasticity is observed for [Ca^2+^]_e_ = 1.3 mM and only LTD for positive and negative timing for [Ca^2+^]_e_ = 1.8 mM (Figure 2). But adjusting the protocol by increasing the pairing frequency from 0.3 to 5–10 Hz, or the number of postsynaptic APs from 1 to 3–4, allows to restore classical Hebbian STDP (Inglebert et al., 2020). These results obtained in young animals need to be confirmed by further studies in the adult and encourage a reexamination of STDP under physiological conditions.

**Table 1:** Selected publications on Spike Timing-Dependent Plasticity.

Reference	Synapses	Induction protocol	Plasticity observed	[Ca^2+^]_e_
Debanne et al. (1994)	CA3-CA1	50–100 pairings @ 0.3 Hz + Postsynaptic burst	LTD (Δt < 0 ms)	2.8 mM
Bi and Poo (1998)	Hippocampal	60 pairings @ 1 Hz + Postsynaptic depolarization	LTP (Δt > 0 ms) and LTD (Δt < 0 ms)	3 mM
	neurons
Markram et al. (1997)	L5-L5	10 pairings @ 20 Hz (2:2 or 5:5 or 10:10)	LTP (Δt > 0 ms)	2 mM
Sjöström et al. (2001)	L5-L5	15 pairings @ 40 Hz (1:5)	LTP (Δt > 0 ms)	2.5 mM
Froemke et al. (2006)	L2/3	60–100 pairings @ 0.2 Hz (1:1)	LTP (Δt > 0 ms) and LTD (Δt < 0 ms	2.5 mM
Froemke et al. (2006)	L2/3	30–40 pairings @ 0.2 Hz (5:5)	No Plasticity, LTD or LTP depending on burst frequency	2.5 mM
Wittenberg and Wang (2006)	CA3-CA1	70–100 pairings @ 0.1–0.5 Hz (1 :1)	LTD only (Δt > 0 ms and Δt < 0 ms)	2 mM
Wittenberg and Wang (2006)	CA3-CA1	100 pairings @ 5 Hz (1:2)	LTD (Δt < 0 ms) and LTP (Δt > 0 ms)	2 mM
Nishiyama et al. (2000)	CA3-CA1	Train of stimuli at 5 Hz + postsynaptic Spike	LTP (*Δt* > 0 ms) and LTD (*Δt* < 0 ms)*	2–2.6 mM
Campanac and Debanne (2008)	CA3-CA1	100 pairings @ 0.3 Hz for LTP	LTD (Δt < 0 ms) and LTP (Δt > 0 ms)	3 mM
		150 pairings @ 0.3 Hz for LTD (1:1)
Pawlak and Kerr (2008)	Corticostriatal	60 pairings @ 0.1 Hz (1:1)	LTD (Δt < 0 ms) and LTP (Δt > 0 ms)	2.5 mM
	pathway
Mishra et al. (2016)	CA3-CA3	300 pairings @ 1 Hz (1:1)	LTP only (Δt > 0 ms and Δt < 0 ms)	2 mM
Inglebert et al. (2020)	CA3-CA1	100 pairings @ 0.3 Hz for LTP	No Plasticity or LTD only	1.3–1.8 mM
		150 pairing @ 0.3 Hz for LTD (1:1)

This table summarizes the plasticity observed in several studies as a function of extracellular calcium concentration. The brain region studied and the induction protocol used are also indicated. The number of repetitions, the pairing frequency and number of pre/post stimulation are specified. For example, 5:5 indicates five presynaptic and five postsynaptic stimulations. *A second LTD window is visible around Δt = 20 ms. STDP, Spike Timing-Dependent Plasticity.

## Reevaluation of STDP Rules

### Multiple STDP Rules

The findings of Inglebert et al. invite a reexamination of plasticity at many synapses. For example, at CA3-CA3 synapses where only LTP is observed (Mishra et al., 2016), it is likely that this would not be the same in physiological calcium. Hebbian STDP (t-LTP for positive timings and t-LTD for negative timings) is found predominantly at excitatory synapses. Despite being ubiquitous, STDP curve can take many different shapes. Anti-Hebbian STDP (t-LTP for negative timings or t-LTD for positive timings) is found largely at inhibitory synapses. In particular, the striatum, which is composed principally of inhibitory neurons, has many different STDP curves depending on the neuron considered (Fino and Venance, 2010). For example, interneurons that expressed Nitric Oxide synthase, present a window of LTP for a positive timing around Δt = +50 ms. In the dorsal cochlear nucleus (DCN), pre-before-post protocol induced LTD in cartwheel cells (Tzounopoulos et al., 2004). Would all these results persist in physiological calcium or plasticity has been overestimated?

### Dendritic Calcium Spikes in Physiological Calcium

Among the factors that could be potentially affected by physiological calcium, the occurrence of dendritic calcium spikes or NMDA spikes that have been shown to be critical in LTP induction (Kampa et al., 2006; Brandalise et al., 2016) could be greatly reduced. A possibility is that in physiological calcium, a larger number of synaptic inputs would be required to trigger an NMDA spike. Alternatively, the spatial extent of the NMDA spike could be reduced in physiological calcium rending induction of plasticity more difficult. Further studies will be required to test these possibilities.

### Calcium Micro-Domains in Physiological Calcium

Calcium micro-domains are supposed to play a critical role in STDP (Mihalas, 2011). A major consequence of reducing external calcium concentration to physiological values is a great reduction of the size of calcium micro-domains. As a consequence, the calcium-sensitive effector might be disconnected from the source of calcium, thus accounting for the observed reduction in plasticity (Inglebert et al., 2020). Additional studies will be, however, required to test further these hypotheses.

### Presynaptic Aspects of STDP in Physiological Calcium

Neocortical STDP relies heavily on presynaptic glutamate release and presynaptic firing rate (Markram et al., 1997; Sjöström et al., 2001, 2003). However, it is now clear that spontaneously released vesicles and evoked transmission use distinct mechanisms (Kavalali, 2015; Abrahamsson et al., 2017). Therefore, physiological calcium could have distinct consequences on evoked and spontaneous release. At many synapses, evoked glutamate release is controlled by pre-synaptic Ca^2+^ entry through voltage-dependent calcium channels (VDCC) and [Ca^2+^]_e_ (Südhof, 2012). Reduced [Ca^2+^]_e_ is associated with decreased synaptic transmission (Borst and Sakmann, 1996; Debanne et al., 1996b; Hardingham et al., 2006). On the contrary, spontaneous release is poorly sensitive to fluctuations in [Ca^2+^]_e_ and is not triggered by Ca^2+^ entry *via* VDCC (Scanziani et al., 1992; Vyleta and Smith, 2011). Interestingly, neocortical presynaptic NMDA receptors regulate both spontaneous and evoked release by distinct molecular pathways (Abrahamsson et al., 2017; Bouvier et al., 2018), are required for neocortical t-LTD (Sjöström et al., 2003) and for hippocampal t-LTD (Andrade-Talavera et al., 2016). The work of Inglebert et al. (2020) focused on the post-synaptic calcium hypothesis but further studies are needed to explore pre-synaptic long-term plasticity.

### Towards Standardization of Induction Protocols

All studies use different protocols (Table 1). The number of repetitions, the pairing frequency, the somato-dendritic distance of the inputs or the number of postsynaptic potentials are different among studies. Therefore, it is often difficult to compare the different results obtained by each study. In physiological calcium (i.e 1.3 mM), it appears that t-LTP and t-LTD requires a greater frequency of pairing (>5 Hz) or a greater number of postsynaptic APs (>3) even with a large number of repetitions (100 or 150; Inglebert et al., 2020). A better understanding of the rules of STDP induction *in vitro* under physiological conditions will allow a more robust application *in vivo*.

### Implication for *In vivo* Exploration of STDP

In opposition to *in vitro* studies, and by definition, *in vivo* studies are inherently in a physiological calcium concentration. First demonstration of STDP *in vivo* was performed in the retinotectal pathway of Xenopus (Zhang et al., 1998). t-LTP and t-LTD were observed but were not robust and easily abolished by hyperpolarizations or spontaneous activities, a limitation recently highlighted by a biophysical model of STDP (Higgins et al., 2014). The use of a non-physiological concentration of calcium may lead to an underestimation of the time scales of memory maintenance as background activities is an important factor for limiting plasticity. Furthermore, most *in vitro* studies are performed in juvenile rodent while *in vivo* studies are performed in older animals. This may constitute an additional limitation to the transposition of the results observed *in vitro*. Several studies suggest that the capacity to induce t-LTD decreases with age (Banerjee et al., 2009; Verhoog et al., 2013) although a recent study has shown t-LTD at cortical layer V synapses in adult mice following pre-before-post protocol (Louth et al., 2021). Similarly, induction of t-LTD by a STDP protocol in the somato-sensory cortex of adult rats by pairing postsynaptic spikes and subthreshold whisker deflection is relatively frequent whereas induction of t-LTP in the same preparation is rare (Jacob et al., 2007). t-LTD disappeared rapidly after a few minutes (5–10 min) and t-LTP was sporadic following pre-before-post pairings (Jacob et al., 2007). As already suggested, STDP in older animals may require protocols that produce stronger depolarization (Meredith et al., 2003). In concordance with this idea, electrical stimulation of afferent input at high frequency paired with post-synaptic burst produced robust t-LTP in cat visual cortex (Frégnac et al., 2010). It is important to note that most of the results observed *in vivo* are obtained in the anesthetized animal. Although there are variations in extracellular calcium concentration of about 0.2 mM between awake and anesthetized animals (Ding et al., 2016), they are not sufficient to produce a major effect on the induction and maintenance of plasticity. An attractive explanation could be that in anesthetized animals, neuromodulation is largely depressed.

### Importance of Neuromodulation

As demonstrated by Inglebert et al. fine tuning of pre- and postsynaptic activity can restore t-LTD and t-LTP in physiological extracellular calcium condition. But would it be possible to restore classic plasticity rules under regular patterns of activity? The key component could be neuromodulation. Interestingly, replay of activity from place-cells with overlapping firing field in hippocampal slices induced t-LTP only in the presence of Carbachol, a cholinergic agonist (Isaac et al., 2009). Many studies have now shown the effects of various neuromodulators on STDP. One of the most explored is dopamine (DA). It is involved in learning and reward processes (Schultz, 1997; Suri and Schultz, 1999). The activation of the D1 receptor (D1-R) has been shown to increase temporal window for t-LTP and to allow induction of t-LTP with fewer spike pairs at glutamatergic synapses of hippocampal neurons (Zhang et al., 2009). At CA3-CA1 synapses, D1-R activation switches t-LTD into t-LTP (Brzosko et al., 2015). In the prefrontal cortex, DA application allow t-LTP induction (He et al., 2015). The effects of the D1-R are widespread (Neve et al., 2004), but many of them could explain the reasons for promoting LTP. They could facilitate signal propagation by inhibiting A-type current (Hamilton et al., 2010; Edelmann and Lessmann, 2011; Yang and Dani, 2014) or simply increased intracellular calcium (Lezcano and Bergson, 2002). D2 receptors (D2-R) are also involved in t-LTP and t-LTD. In lateral amygdala, D2-R gates LTP induction by suppressing feedforward inhibition (Bissière et al., 2003). In some cases, a synergy is observed between the two receptors. In layer V of the prefrontal cortex, D1-R and D2-R co-activation enables the induction of t-LTP at extended timing interval (Xu and Yao, 2010). Noradrenaline (NA) is also involved in memory formation. Activation of β-adrenergic receptors (β-R) increases intracellular calcium (Seol et al., 2007) and facilitate bAP by inhibition of A-type current (Yuan et al., 2002) or SK channels (Faber et al., 2008). At CA3-CA1 synapses, β-R activation increases the temporal window induction for t-LTP (Lin et al., 2003). In layer II/III of visual cortex, co-activation of β-R and α-adrenergic receptor (α-R) are required for bidirectional STDP in fast-spiking and somatostatin interneurons. β-R activation promoted t-LTP whereas α-R activation induced t-LTD (Huang et al., 2013, 2014). Neuromodulation could be seen as the necessary factor for the induction of t-LTP and t-LTD in physiological calcium without tuning pre- and postsynaptic activity.

## Conclusion

### Use of Physiological Calcium Levels for Studying Short-Term Synaptic Plasticity

The use of physiological external calcium concentration not only modulates the learning rules for long-term synaptic plasticity but it also enhances context-dependent synaptic plasticity. Analog-digital modulation of action potential-evoked synaptic transmission lies on modification of spike shape, by either broadening the axonal spike (Shu et al., 2006; Kole et al., 2007) or by modulating its amplitude (Rama et al., 2015; Zbili et al., 2020). Because transmitter release is almost maximal in high calcium, conditions that enhance release are somehow difficult to reach. Indeed, switching to physiological calcium concentration (i.e., 1.3 mM) was found to significantly enhance spike amplitude-dependent synaptic plasticity (Rama et al., 2015; Zbili et al., 2020).

### Use of Physiological Calcium Levels for Studying Intrinsic Plasticity

Hebbian plasticity and Intrinsic plasticity are closely linked and are synergistically modified (Debanne et al., 2019). Generally, t-LTP is associated with an increase in excitability and t-LTD with a reduced excitability (Ganguly et al., 2000; Li et al., 2004). Interestingly, recordings using physiological calcium show a significant increase excitability of CA1 pyramidal neurons: lowered firing threshold, increased spontaneous firing and more depolarized resting membrane potential (Bjorefeldt et al., 2018). Dendritic integration or EPSP-Spike coupling is also modified with synaptic changes (Campanac and Debanne, 2008). Generally, LTP is accompanied by an increase in the probability of emitting an AP for the same synaptic input (EPSP-spike potentiation) whereas LTD is accompanied by a decrease in the probability of emitting an AP (EPSP-spike depression). This plasticity of the output, without modification of the input, result partially from postsynaptic changes in voltage dependent channels such as I_H_ or I_A_ (Daoudal and Debanne, 2003). EPSP-Spike coupling modification is also conditioned by changes in inhibitory synaptic transmission. Intriguingly, recording in physiological calcium in hippocampus have revealed that excitatory-inhibitory balance was disrupted and disynaptic inhibition was strongly decreased (Aivar et al., 2014).

## Author Contributions

YI and DD wrote the article and YI built the figures. All authors contributed to the article and approved the submitted version.

## Conflict of Interest

The authors declare that the research was conducted in the absence of any commercial or financial relationships that could be construed as a potential conflict of interest.

## Publisher’s Note

All claims expressed in this article are solely those of the authors and do not necessarily represent those of their affiliated organizations, or those of the publisher, the editors and the reviewers. Any product that may be evaluated in this article, or claim that may be made by its manufacturer, is not guaranteed or endorsed by the publisher.
